# Diagnostic Accuracy of Coronary CT Angiography in Ruling Out Significant Coronary Artery Disease in Candidates for Transcatheter Aortic Valve Replacement

**DOI:** 10.3390/jcdd12100395

**Published:** 2025-10-06

**Authors:** Chiara Gallo, Alfonso Campanile, Carmine Izzo, Sonia Paoletta, Valentina Russo, Pierpaolo Chivasso, Francesco Vigorito, Marco Di Maio, Michele Ciccarelli, Amelia Ravera, Tiziana Attisano, Giuliano Maraziti, Davide Di Gennaro, Enrico Coscioni, Carmine Vecchione, Oliviero Caleo

**Affiliations:** 1 Department of Advanced Biomedical Sciences, Federico II University Hospital, 80131 Naples, Italy; chiara.gallo6@studenti.unina.it; 2 Intensive Cardiac Care Unit, University Hospital “San Giovanni di Dio e Ruggi d’Aragona”, 84131 Salerno, Italy; alfonso.campanile@sangiovannieruggi.it (A.C.); amelia.ravera@sangiovannieruggi.it (A.R.); 3 Department of Cardiology, University Hospital “San Giovanni di Dio e Ruggi d’Aragona”, 84131 Salerno, Italy; 4 Department of Medicine, Surgery and Dentistry, University of Salerno, 84081 Baronissi, Italy; v.russo43@studenti.unisa.it (V.R.); mciccarelli@unisa.it (M.C.); cvecchione@unisa.it (C.V.); 5 Department of Radiology, University Hospital “San Giovanni di Dio e Ruggi d’Aragona”, 84131 Salerno, Italy; sonia.paoletta@sangiovannieruggi.it (S.P.); giuliano.maraziti@sangiovannieruggi.it (G.M.); oliviero.caleo@sangiovannieruggi.it (O.C.); 6 Department of Cardiac Surgery, University Hospital “San Giovanni di Dio e Ruggi d’Aragona”, 84131 Salerno, Italy; pierpaolo.chivasso@sangiovannieruggi.it (P.C.); enrico.coscioni@sangiovannieruggi.it (E.C.); 7 Interventional Cardiology Unit, University Hospital “San Giovanni di Dio e Ruggi d’Aragona”, 84131 Salerno, Italy; francesco.vigorito@sangiovannieruggi.it (F.V.); marco.dimaio@sangiovannieruggi.it (M.D.M.); tiziana.attisano@sangiovannieruggi.it (T.A.); 8 Department of Radiotherapy, University Hospital “San Giovanni di Dio e Ruggi d’Aragona”, 84131 Salerno, Italy; davide.digennaro@sangiovannieruggi.it

**Keywords:** aortic stenosis, coronary computed tomography angiography (cCTA), transcatheter aortic valve implantation (TAVI), coronary artery disease (CAD), invasive coronary angiography (ICA), cardiovascular disease

## Abstract

Obstructive coronary artery disease (CAD) is common in patients undergoing transcatheter aortic valve implantation (TAVI). While invasive coronary angiography (ICA) is the gold standard for coronary evaluation, coronary computed tomography angiography (cCTA) is gaining interest for its potential to exclude obstructive CAD during pre-procedural imaging. This study aimed to assess the diagnostic accuracy of cCTA in ruling out significant CAD in TAVI candidates. We retrospectively analyzed 95 TAVI candidates (mean age 77.7 ± 8.5 years) who underwent both cCTA and ICA. Diagnostic performance of cCTA—sensitivity, specificity, positive predictive value (PPV), negative predictive value (NPV), and accuracy—was assessed using ICA as the reference, in both patient- and vessel-based models. Obstructive CAD was defined as ≥50% luminal stenosis or occlusion of a stent/bypass graft. ICA detected obstructive CAD in 27 patients (28.4%). Excluding non-evaluable cases, cCTA showed a negative predictive value (NPV) of 97% (patient-level) and 95% (vessel-level), with a diagnostic accuracy of 85% and 87%, respectively. Including all patients, regardless of scan quality, the NPV remained high (97%), although overall accuracy dropped to 67% (patient-level) and 66% (vessel-level). cCTA demonstrated high accuracy in excluding significant CAD, with a stable NPV of 95–97%. The relatively high rate of non-diagnostic scans and the single-center, retrospective design suggest that its role should be considered complementary to ICA, potentially reducing—but not replacing—the need for ICA in selected TAVI candidates.

## 1. Introduction

Transcatheter aortic valve implantation (TAVI) has significantly changed the management of patients affected by severe aortic stenosis since its introduction in 2002 [1]. According to the European guidelines, TAVI represents the procedure of choice (class IA) in patients older than 75 years or at high risk (STS-Prom/EuroSCORE > 8) [2,3]. However, not every patient who refused or is at high risk for surgery is a good candidate for TAVI due to specific technical and anatomic criteria that must be met to ensure a high successful rate to the procedure. Therefore, the role of non-invasive imaging findings becomes crucial in patient selection for TAVI [4]. In particular, cardiac computed tomography angiography (CTA) is now essential for an accurate anatomical characterization of the aortic root and vascular accesses, which contributes to a significant reduction in peri-procedural complications and mortality [5]. In addition, due to the not negligible percentage of patients presenting coronary artery disease (CAD) on top of severe aortic stenosis (from 38% to 75%) [6,7], the potential application of coronary CTA (cCTA) is gaining increasing interest as a way to avoid additional invasive assessments by means of coronary angiography in such a frail category of patients [8]. Currently, invasive coronary angiography (ICA) is still the gold standard for coronary assessment in patients undergoing TAVI [9], and despite uncertainty as to its clinical effectiveness, coronary revascularization is still recommended in the case of significant proximal lesions [10,11]. Indeed, current guidelines endorse cCTA as an alternative to ICA prior to TAVI only in patients with a low pre-test probability of obstructive CAD [2]. Nonetheless, previous studies have investigated the potential application of cCTA in coronary assessment in patients undergoing TAVI, finding potential space for its systematic application in this context [12,13]. Due to the significant advancement in technologies, in comparison to the cited studies, we aimed to explore the feasibility of cCTA in ruling out significant CAD in candidates for TAVI, using one of the latest-generation wide-detector scanners.

## 2. Materials and Methods

### 2.1. Study Design and Population

**Figure 1:**
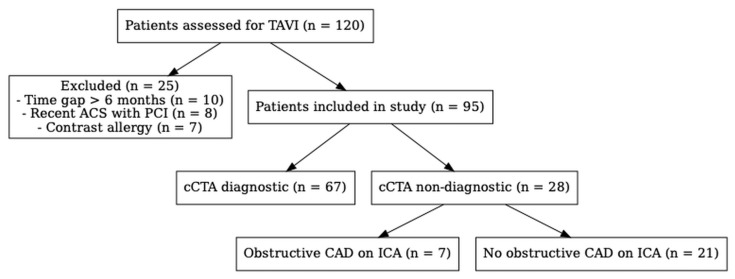
Flowchart of patient selection and diagnostic evaluability. A total of 120 patients were screened for TAVI between June 2022 and May 2024. After exclusions, 95 were included in the final analysis. Coronary CTA was non-diagnostic in 28 patients due to factors including stents, heavy calcification, motion artifacts, or poor contrast opacification. Of these, 7 were ultimately diagnosed with obstructive CAD with invasive coronary angiography.

All patients admitted to our hospital (University Hospital of Salerno) with an indication for the TAVI procedure due to severe aortic stenosis between June 2022 and May 2024 were retrospectively enrolled in our study. In the whole cohort, the presence of CAD was investigated using both cCTA and ICA before the TAVI procedure. Exclusion criteria were as follows: previous severe adverse reactions to an iodinated contrast agent, TAVI candidates with a time interval greater than six months between cCTA and ICA, and a recent acute coronary syndrome (in the last 12 months) treated by means of coronary angioplasty with stent implantation. We included patients with estimated glomerular filtrate rate (eGFR) < 30 mL/min, patients with previous history of aortic valve replacement (therefore candidates for a valve in valve procedure), and patients with a background history of coronary revascularization (previous coronary artery bypass and/or coronary angioplasty). We also included patients with poor image quality and patients with cCTA performed after the ICA (always within six months) (Figure 1). Although this was a retrospective study, our institution (due to its university nature) routinely obtains written informed consent from all patients undergoing CT imaging as well as all other exams and procedures, which includes permission for the anonymized use of clinical and imaging data for research purposes. This practice is part of our standard institutional policy and does not affect patient care or eligibility for procedures. Patients who decline consent are excluded from research use but still undergo CT as part of their clinical care. The cohort analyzed in this study therefore represents all consecutive patients who provided such consent during the study period.

### 2.2. CT Scan Protocol

CTA was performed using a 16 cm wide-detector, 256-slice scanner (Revolution CT; GE Healthcare, Milwaukee, WI, USA), without any patient medical preparation (no administration of nitrates or beta-blockers). CT examinations were performed using the following parameters: detector geometry: 256 rows with 832 detection elements per row; slice thickness: 0.625 mm; portal rotation time: 280 ms; prospective triggering; iterative reconstruction algorithm (DLIR; GE Healthcare). The X-ray tube voltage was set to 120 kV with automatic milli-amperage regulation.

At the beginning, scout radiographs of the chest and abdomen-pelvis were acquired in antero-posterior and lateral projections.

Before contrast medium injection, an ECG-gated scan was performed, covering the entire heart, from the tracheal bifurcation to the cardiac apex, in order to calculate the calcium score, i.e., quantification of valve calcification.

During the contrast phase, a bolus of 80 mL of high-concentration iodinated contrast (370 mg/mL iopromide; Ultravist, Bayer, Leverkusen, Germany) was injected at a rate of 5 mL/s into an antecubital vein (using a 20 G or larger cannula), preceded and followed by a 30 mL saline flush.

Correct scan timing was achieved using the bolus tracking technique, placing a region of interest (ROI) on the ascending aorta with a threshold of 150 HU.

The angiographic phase was performed in a single apnea through positioning of three consecutive and linked axial volumes, so a spiral acquisition of the upper thorax was followed by a 16 cm prospective ECG-gated scan of the heart, followed by an additional spiral scan covering the abdominal aorta up to the iliac–femoral axes.

A prospective ECG-gated scan was acquired to cover a cardiac cycle’s phase between 20 and 90%, retrospectively reconstructed with 10% intervals, in order to obtain an accurate evaluation of the aortic annulus in telesystolic phase and sufficient data for coronary artery assessment.

The exam was completed by an additional non-ECG-gated spiral thoraco-abdominal scan, performed 40 s after the angiographic phase.

### 2.3. Image Reconstruction and Data Analysis

Images were post-processed and interpreted by an experienced radiologist on a dedicated workstation (AW Server 3.2 EXT 4.0), using axial images, multiplanar reconstructions, and maximum intensity projection (MIP) where necessary. The Agatston method was used for detecting the calcium score.

Subsequently, images were reconstructed in the telesystolic phase (30%) using a dedicated pre-TAVI planning application to assess the dimensions and shape of the aortic annulus, aortic valve anatomy, distance between the annulus and coronary ostia, height of the sinotubular junction, bulb diameters, dimensions and state of the ascending aorta, as well as dimensions and state of the iliac–femoral arterial axes. Then, detailed reports were obtained and sent to PACS.

**Figure 2:**
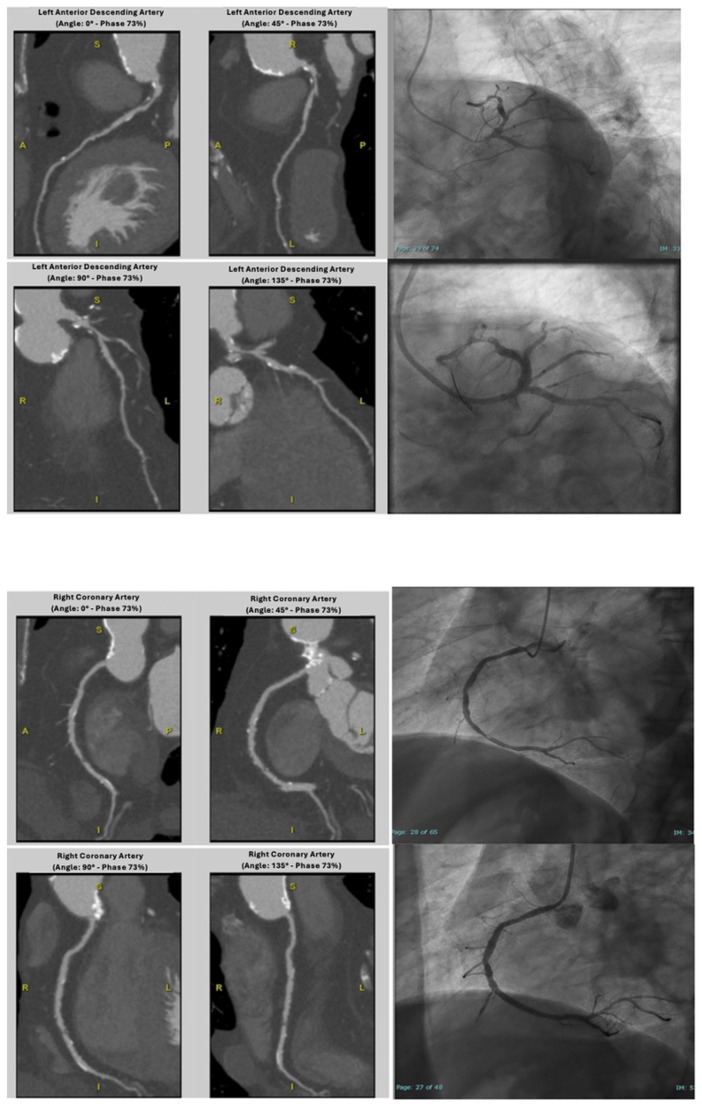
CT reconstructions of coronary arteries and angiographic findings before and after PTCA. On CTA: short common trunk with calcified plaque causing a 40–50% stenosis. At the level of the proximal LAD, fibrolipidic plaque with severe stenosis (>70%; minimal luminal area of approximately 1 mm^2^). RCA with diffuse atherosclerosis and focal fibrolipidic plaque in its distal segment causing subocclusive luminal stenosis. These alterations were confirmed by an angiographic exam and treated with PTCA. PTCA = Percutaneous Transluminal Coronary Angioplasty, LAD = Left Anterior Descending artery, RCA = Right Coronary Artery.

After visually identifying the optimal diagnostic phases, coronary arteries were evaluated through a dedicated software; images were processed with the “Snap Shot Freeze technique” to reduce motion artifacts (Figure 2). Coronary arteries were segmented as recommended by the AHA guidelines [14] and analyzed using multiplanar reconstructions accompanied by measurements of the vessel area in the case of luminal stenosis. Sharp-kernel reconstruction was available and applied to improve accuracy in the diagnostic phase in the presence of severe calcification, in order to reduce blooming artifacts. ICA, on the other hand, was performed by experienced cardiologists at the Hemodynamics Department, using standard techniques and projections. All procedures were conducted in accordance with current guidelines and regulations.

## 3. Statistical Analysis

Statistical analysis was conducted using SPSS software version 25.0 (SPSS Inc., Chicago, IL, USA) and R software, version R.4.0.5 (R Foundation for Statistical Computing, Vienna, Austria).

Continuous variables were expressed as mean ± standard deviation (SD), and discrete variables were expressed as absolute numbers and percentages.

Diagnostic performance of cCTA was expressed as sensitivity, specificity, positive predictive value (PPV), negative predictive value (NPV), and accuracy, with corresponding 95% confidence intervals, using ICA results as the reference standard, both in a vessel- and patient-based model.

Obstructive CAD was defined as the presence of any plaque causing, in both cCTA and ICA, a luminal stenosis ≥ 50% in the epicardial coronary vessel (since mild stenosis typically does not require further diagnostic workup), or as occlusion of a previously implanted stent or bypass graft.

In the vessel-based analysis, if more than one obstructive stenosis was present in the same vessel, the most severe lesion was considered representative of the vessel.

cCTA was performed after ICA in 51 patients; therefore, to assess the likelihood of potential bias from the fact that CTA readers were not necessarily blinded to ICA results, we performed a sensitivity analysis excluding these patients from the analysis.

## 4. Results

The study population’s characteristics are reported in Table 1. A total of 95 patients were enrolled. The mean age was 77.7 ± 8.5 years, and the female gender was adequately represented (52.6% of the whole study cohort). A high prevalence of patients with hypertension (73.7%) and dyslipidemia (62%) was observed. The mean heart rate during the CT examination was 77.8 ± 24.6 bpm, with 26.3% of patients showing atrial fibrillation. The mean dose-length product (DLP) was 1280.64 ± 630.05 mGy·cm, corresponding to an estimated effective dose of 19.2 ± 9.45 mSv.

Overall, 27 patients (28.4%) had obstructive CAD on ICA. These patients were more often male, older than 75 years, and showed a high comorbidity burden, with coexistence of two or more cardiovascular risk factors and a higher calcium score (Table 2). The prevalence of one-, two-, or three-vessel disease was 59%, 11%, and 30%, respectively.

### 4.1. Coronary CTA Findings in Patients-Based Analysis

cCTA was judged as non-diagnostic in 28 cases (29.5%) due to the presence of previously implanted stents, extensive vascular calcifications, significant heart-rate-related artifacts, or suboptimal vessel opacification. A detailed breakdown of all causes is provided in Table 3. Among these 28 patients with non-diagnostic cCTA, ICA identified obstructive stenoses in seven patients. Aiming to show clear and complete findings as much as possible, we decided to report diagnostic performance both excluding and including non-diagnostic scans. Indeed, while the exclusion process improves accuracy metrics, the inclusion of the whole cohort better reflects the real-world performance.

**Figure 3:**
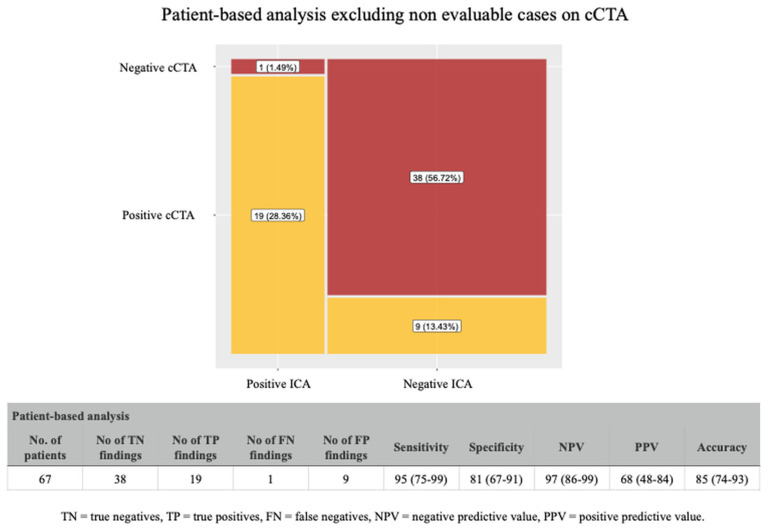
Diagnostic performance of cCTA (patient-based analysis) excluding non-diagnostic cases. In patients with diagnostic-quality cCTA, the method demonstrated a positive predictive value of 68%, a negative predictive value of 97%, and an overall accuracy of 85% compared to ICA.

**Figure 4:**
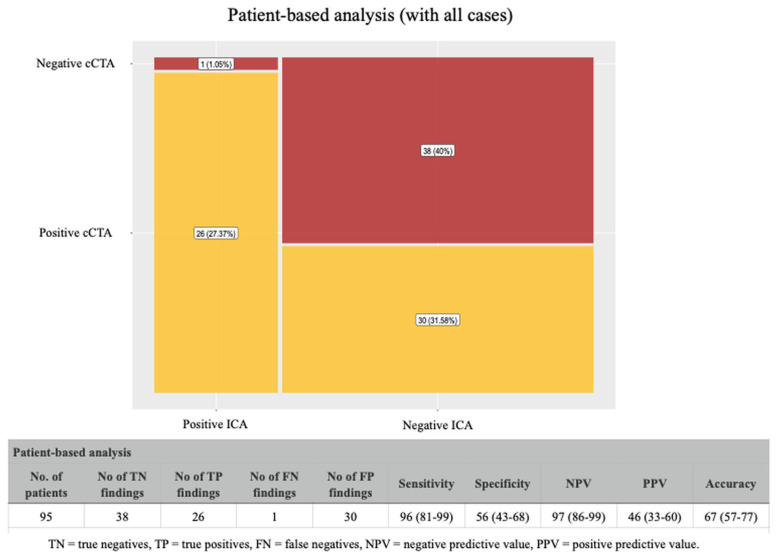
Diagnostic performance of cCTA (patient-based analysis) including non-diagnostic cases. When non-diagnostic cCTA scans were included in the analysis, the positive predictive value dropped to 46% and overall accuracy to 67%, while the negative predictive value remained high at 97%.

When we excluded all the non-diagnostic cases, cCTA showed a positive predictive value of 68%, a negative predictive value of 97%, and an overall accuracy of 85% (Figure 3). As expected, the analysis including the non-diagnostic cases (Figure 4), showed a significant reduction in the positive predictive value (46%) and in the overall accuracy (67%); however, the negative predictive value was still very high (97%).

### 4.2. Coronary CTA Findings in Vessel-Based Analysis

**Figure 5:**
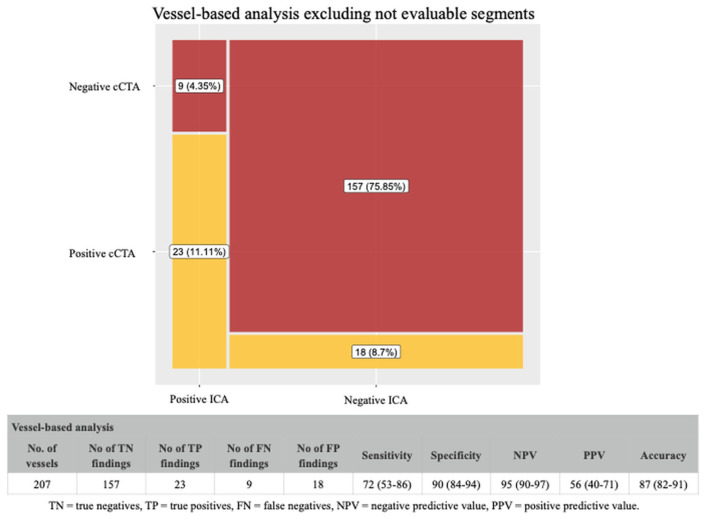
Diagnostic performance of cCTA (vessel-based analysis) excluding non-evaluable vessels. After excluding 78 non-evaluable vessels, cCTA showed a positive predictive value of 56%, a negative predictive value of 95%, and an overall accuracy of 87% in identifying obstructive CAD at the vessel level.

**Figure 6:**
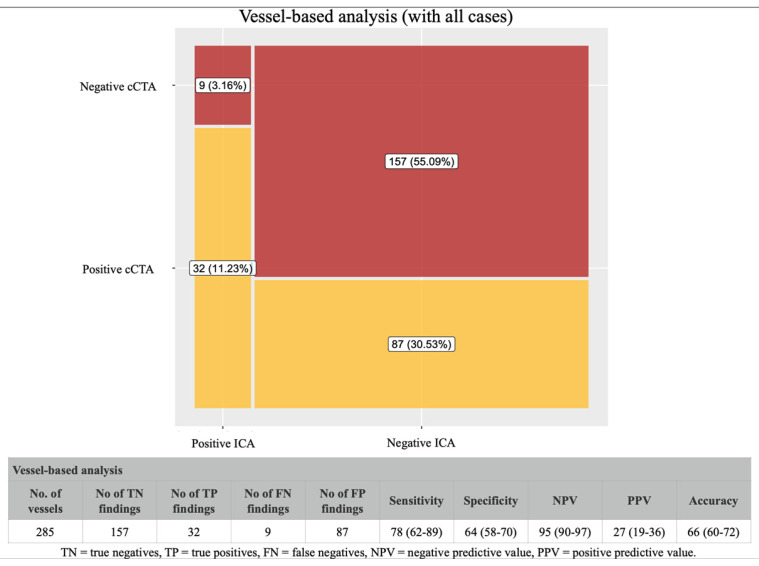
Diagnostic performance of cCTA (vessel-based analysis) including non-evaluable vessels. Including non-evaluable vessels as positive findings led to a reduction in positive predictive value (27%) and total accuracy (66%), while the negative predictive value remained at 95%.

A total of 285 vessels were examined, and 78 were classified as not evaluable. When we excluded non-evaluable vessels from the analysis, the cCTA showed a positive predictive value of 56%, a negative predictive value of 95%, and a global accuracy of 87% (Figure 5). The inclusion of non-evaluable segments in the analysis (Figure 6) led to a significant reduction in the positive predictive value and total accuracy (to 27% and 66%, respectively), while the negative predictive value was still consistently high (95%).

### 4.3. Coronary CTA Findings After Sensitivity Analysis

To assess the impact on the final results interpretation of a potential diagnostic review bias due to the retrospective study design and the fact that CTA readers were not necessarily blinded to ICA results, we performed a sensitivity analysis excluding the 51 patients on whom cCTA was performed after ICA, aiming to reduce, at least, the “blinding” issue. The results, in this specific subgroup of patients, (Supplementary Figures S1–S4) demonstrated that key diagnostic performance measures remained consistent in comparison to the primary analysis, reinforcing, therefore, the robustness of our findings.

## 5. Discussion

Two main results emerge from our study: (1) in an unselected cohort of candidate patients for TAVI, coronary CT was feasible in 2/3 of patients (non-diagnostic cases around 29.5% in our analysis); (2) cCTA showed high accuracy in excluding the pathology, with a stable negative predictive value around 95–97%, showing the potentiality of avoiding ICA in approximately 40% of patients. Nonetheless, given that nearly one-third of scans were non-diagnostic, these findings must be interpreted cautiously. Our results support a selective rather than a universal substitution of ICA by cCTA.

As to the first point, different studies have focused the attention on the diagnostic performance of coronary CT in the pre-TAVI setting, indicating that even with some limitation due to the advanced age of this specific population, the high prevalence of calcified plaques, and the coexistence of comorbidities such as atrial fibrillation, the procedure is feasible, especially when latest-generation scanners with upgraded protocols and software are used [12,13,15,16,17]. In particular, in the study by Annoni et al., the authors showed that using a third-generation CT scanner with wide scan coverage along the patient Z-axis and a 16 cm detector coverage, they were able to perform a scan of the whole heart volume during one heartbeat (0.8 s scan time), reducing therefore the potential artifacts related to heart rate or movement, even in the case of patients with AF [12]. Similarly, a series of studies have shown how the development of dual-source technology generates an improvement in the diagnostic accuracy of CT for the evaluation of the coronary arteries in the pre-TAVI study by increasing temporal solutions [18]. In our cohort, the non-diagnostic case rate of 29.5% looks reasonable when compared with the above cited studies. Indeed, the rate of non-diagnostic/poor-quality cCTA cases of the aforementioned studies, also taking into account differences in definition of the cCTA imaging quality, and in populations analyzed, ranged from 4.3 to 57.5%. Our non-diagnostic rate of 29.5% underscores a key limitation in applying cCTA broadly in the TAVI population. This reflects technical challenges including extensive calcification, prior stenting, and atrial fibrillation, all common in this elderly cohort. Therefore, although diagnostic performance was good in evaluable cases, clinical implementation must anticipate that a substantial subset of patients will still require ICA.

Regarding the second point, the diagnostic performance of coronary CT in our study was comparable to what had been detected in previous studies [12,13,16,19,20]. Indeed, in all these studies, the positive and negative predictive values ranged, respectively, from 41.3 to 80.6%, and from 91.3 to 100%, resulting, therefore, comparable to what we detected in our cohort—PPV: 46%; NPV: 97% [12,13,16,19,20]. Currently, cCTA is considered a reasonable method in selected patients with low or moderate pre-test probability of obstructive CAD, and in some cases, it can serve as a non-invasive alternative to ICA to rule out significant CAD. However, it does not fully substitute ICA, especially in patients with high calcium burden, prior revascularization, or non-diagnostic scans [21]. Our study, performed in an unselected population with a not negligible cardiovascular comorbidity burden, highlights a potential wider application of this method in order to avoid invasive coronary angiography in a significant percentage of patients (in our case, almost 40% of patients with negative results on cCTA were confirmed as not affected by obstructive CAD on ICA). One of the main reason of these results is related to the technology advancement achieved in recent years such as the application of a wide-detector scanner, which allows the acquisition of the entire cardiac volume in a single beat, and the increased rotation speed of the X-ray tube with a significant improvement in temporal resolution [22].

Another important technical aspect is related to a proper “freezing” of cardiac motion. The “Snapshot Freeze (SSF)” technique, used in our study, utilizes information from adjacent cardiac phases within a single cardiac cycle to characterize coronary arteries’ motion, analyzing their path and their speed of movement caused by heartbeat, and to compensate for the motion. It uses these data to calculate and correct coronary arteries’ position in the target phase, thus improving the sharpness and diagnostic quality of images [23]. This algorithm works per vessel and per segment, correcting coronary arteries’ motion for each voxel. In other words, the system “virtually freezes” heart motion during the scan. Since this technique characterizes coronaries’ motion within a single cardiac cycle, it is less affected by factors such as arrhythmia, and it is more effective in patients with complex conditions. For SSF reconstruction, at least two or three additional phases are required; although this increases the radiation dose, the image quality improves significantly [24,25].

Despite promising results, the extensive use of cCTA in the pre-TAVI setting is still limited by the presence of a modest positive predictive value (68% in our case), meaning that, in a certain percentage of cases, cCTA identifies significant CAD not subsequently confirmed by ICA. This is even more true when non-evaluable coronary segments are included, as positive, in the analysis, which causes a significant reduction in overall accuracy. Therefore, the results in the whole cohort, which have been presented in order to better reflect the real-word performance of cCTA, emphasize the important concept of the quality of technology equipment available before thinking of a systematic implementation of this method in clinical practice. However, we wish to underline that even if the presence of non-diagnostic cases still represents an undoubtful limit of cCTA, shared, as already discussed, with several different studies, the cCTA contribution in clinical practice looks beneficial due to the absence of additional risks for patients as the coronary artery study is obtained from the same CT scan foreseen in the pre-TAVI diagnostic-therapeutic planning [3,17,20,21]. Moreover, the emergence of photon-counting CT and advanced reconstruction algorithms may help reduce the rate of non-diagnostic exams in future practice [26,27]. Although NPV was consistently high, the overall accuracy of 85–87% implies that a non-negligible number of patients could be misclassified. Extrapolated to higher-volume centers, this could translate into clinically relevant numbers of false negatives and false positives, highlighting the importance of confirmatory strategies and careful patient selection.

## 6. Study Limitations

Our study has several limitations that need to be accounted in order to better interpret the final results. First, this is a retrospective study, conducted in a single center with a modest sample size. These factors, along with potential institutional biases, strongly limit the generalizability of our findings and necessitate external validation in larger multicenter cohorts. Second, interventional cardiologists and radiologists were sometimes aware, beforehand, of the results of the ICA or cCTA. However, our sensitivity analysis, despite showing expected modifications in accuracy values, demonstrated a certain consistency of results compared to the primary analysis, supporting, therefore, the internal validity of our findings. Despite that, the lack of blinding between radiologists and cardiologists interpreting cCTA and ICA findings remains a major limitation which might be overcome only in future prospective studies. Third, our data concerning CT diagnostic performance in vessels with stents and CABG are limited to a small number of patients, and this could have influenced the accuracy of our results. Fourth, a recognized limitation of cCTA is the lack of functional assessment of coronary lesions, which is increasingly used during ICA via fractional flow reserve (FFR) to guide revascularization decisions. Recent developments in computational methods such as Quantitative Flow Ratio (QFR) or FFR-CT allow non-invasive functional assessment based on cCTA datasets. Although not available in our institution at the time of data collection, future studies integrating QFR into the cCTA protocol may enhance diagnostic precision and clinical applicability, particularly by improving PPV and avoiding unnecessary ICA or PCI [28]. The ongoing debate about the prognostic benefit of revascularization before TAVI, and the recent RCTs suggesting possible revascularization benefit in some subsets, further complicate the role of cCTA as a sole gatekeeper. This reinforces the need for a tempered interpretation of our findings.

Finally, no follow-up data are available, limiting therefore any discussion about the potential harm of conducting an unnecessary ICA.

## 7. Conclusions and Future Perspectives

In our study, we showed that a coronary CT, performed with a wide detector, is not only feasible but also able to achieve a high negative predictive value in ruling out obstructive CAD, representing a promising non-invasive adjunct to traditional ICA in carefully selected patients. Larger multicenter prospective studies are needed before considering systematic replacement of ICA. Avoiding invasive angiography can indeed potentially reduce the complications related to an invasive approach and reduce the risk of contrast-induced nephropathy, reducing the total amount of contrast medium for each single patient [29].

Despite technological advancements, some diagnostic limitations of cCTA remain, particularly in the presence of severe calcifications or stents. However, the application of advanced algorithms such as Snapshot Freeze and the continuous development of photon-counting technologies suggest that these issues will be further reduced in the near future. Looking ahead, the integration of cCTA into the routine pre-TAVI assessment pathway may significantly reshape clinical workflows, especially as scanner technology and post-processing algorithms continue to evolve. Future prospective, multicenter studies are warranted to confirm the diagnostic reliability of cCTA across broader patient populations, particularly those with high coronary calcium scores or prior revascularization. Moreover, evaluating the cost-effectiveness of a cCTA-first approach in reducing the need for invasive coronary angiography—along with its potential to minimize contrast exposure, procedural delays, and complications—will be crucial in supporting guideline revisions. With continued innovation in CT imaging, including the implementation of photon-counting detector technology and enhanced motion correction algorithms, the role of cCTA is poised to expand well beyond selected low-risk cohorts, potentially becoming a standard, non-invasive gatekeeper of ICA in the majority of TAVI candidates.

## Figures and Tables

**Table 1:** Study population characteristics.

Total Number of Patients	95
Age (years)	77.7 ± 8.5
Male/female	45/50
BMI (kg/m^2^)	26.6 ± 4.6
BSA (m^2^)	1.73 ± 0.22
Baseline creatinine (mg/dL)	1.35 ± 1.05
Base hemoglobin (g/dL)	11.8 ± 1.8
Heart rate (bpm)	77.8 ± 24.6
EF (%)	49 ± 10.8
EF < 50%, n (%)	37 (39)
Aortic valve mean gradient (mmHg)	38.7 ± 14.9
CAC score	1972.5 ± 1425.3
Atrial fibrillation, n (%)	25 (26.3)
Patients with previous coronary stenting, n (%)	13 (13.7)
Stent number	27
Drug Eluting Stent (DES), n	27
Patients with previous CABG, n (%)	9 (9.5)
Patients with a background history of aortic valve replacement, n (%)	8 (8.4)
Cardiovascular risk factors	
Hypertension, n (%)	70 (73.7)
Dyslipidemia, n (%)	59 (62.1)
Diabetes mellitus, n (%)	40 (42.1)
Current smoking, n (%)	28 (29.5)
Family history of CAD, n (%)	7 (7.4)

BMI: body mass index; BSA: body surface area; CABG: coronary arteries bypass grafting; CAD: coronary artery disease; EF: ejection fraction; CAC score: coronary artery calcium score.

**Table 2:** Main characteristics of patients with obstructive CAD on ICA (N = 27).

Patients with Two or More Cardiovascular Risk Factors; N (%)	20 (74.1)
Male sex, n (%)	17 (63)
Age ≥ 75 years, n (%)	16 (59.3)
High calcium score (>1200 for women and >2000 for men), n (%)	13 (48.1)
BMI ≥ 25, n (%)	13 (48.1)

**Table 3:** Causes of non-diagnostic coronary CT angiography (cCTA) scans (n = 28).

Cause of Non-Diagnostic Scan; N (%)	28 (29.5)
Extensive coronary calcification, n (%)	11 (39.3%)
Previously implanted coronary stents, n (%)	8 (28.6)
Motion artifacts (e.g., atrial fibrillation), n (%)	5 (17.9)
Poor contrast opacification, n (%)	3 (10.7)
Image noise due to high BMI or artifacts, n (%)	1 (3.5)

## Data Availability

The data presented in this study are available on request from the corresponding author due to privacy.

## Supplementary Materials

The following supporting information can be downloaded at: https://www.mdpi.com/article/10.3390/jcdd12100395/s1. Figure S1: Diagnostic performance of cCTA (patient-based analysis) after exclusion of both patients in whom cCTA was performed after ICA and with non-diagnostic cCTA. In this subgroup of 35 patients, results remained consistent with the main analysis, confirming robustness of primary findings. Figure S2: Diagnostic performance of cCTA after exclusion of patients on whom cCTA was performed after ICA, but including also non-diagnostic cases. As in the primary analysis, the inclusion of non-diagnostic cCTA scans caused a sensible reduction in overall accuracy and PPV, while maintaining a high NPV. Figure S3: Diagnostic performance of cCTA (vessel-based analysis excluding non-evaluable segments) in the sensitivity cohort (with exclusion of patients on whom cCTA was performed after ICA). Performance metrics were comparable to the primary analysis, with high NPV and acceptable accuracy after excluding non-evaluable coronary segments. Figure S4: Diagnostic performance of cCTA (vessel-based analysis including non-evaluable segments) in the sensitivity cohort (with exclusion of patients on whom cCTA was performed after ICA). As in the full cohort, inclusion of non-evaluable vessels diminished PPV and accuracy, reinforcing the importance of careful interpretability criteria in clinical application.
