# Supplementary material for: Diagnostic Accuracy of Coronary CT Angiography in Ruling Out Significant Coronary Artery Disease in Candidates for Transcatheter Aortic Valve Replacement
Source: J Cardiovasc Dev Dis. 2025 Oct 6;12(10):395. doi: 10.3390/jcdd12100395 (PMC12564097; doi:10.3390/jcdd12100395)
Supplement: Supplementary file 1 [file jcdd-12-00395-s001.zip › jcdd-3832867-supplementary.pdf]

Journal of
Cardiovascular Development and Disease

Patient-based sensitivity analysis
(excluding patients with cCTA performed after invasive angiography and with not evaluable segments)

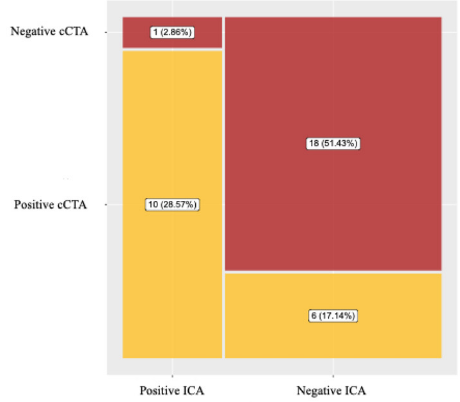

Patient-based analysis									
No. of patients	No of TN findings	No of TP findings	No of FN findings	No of FP findings	Sensitivity	Specificity	NPV	PPV	Accuracy
35	18	10	1	6	91 (59-100)	75 (53-90)	95 (74-100)	62 (35-85)	80 (63-92)

TN = true negatives, TP = true positives, FN = false negatives, NPV = negative predictive value, PPV = positive predictive value.

Supplementary Figure 1. Diagnostic performance of cCTA (patient-based analysis) after exclusion of both patients in whom cCTA was performed after ICA and with not diagnostic cCTA. In this subgroup of 35 patients results remained consistent with the main analysis, confirming robustness of primary findings.

Patient-based sensitivity analysis (excluding patients with cCTA performed after invasive angiography)

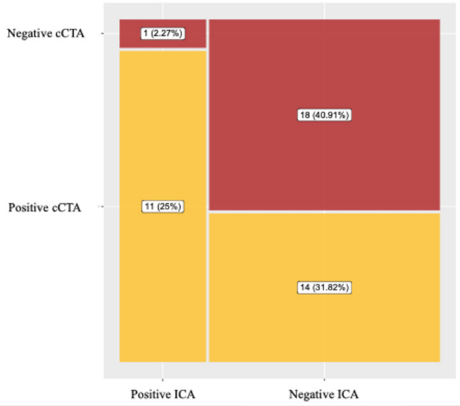

Patient-based analysis									
No. of patients	No of TN findings	No of TP findings	No of FN findings	No of FP findings	Sensitivity	Specificity	NPV	PPV	Accuracy
44	18	11	1	14	92 (62-100)	56 (38-74)	95 (74-100)	44 (24-65)	66 (50-79)

TN = true negatives, TP = true positives, FN = false negatives, NPV = negative predictive value, PPV = positive predictive value.

Supplementary Figure 2. Diagnostic performance of cCTA after exclusion of patients in whom cCTA was performed after ICA, but including also non-diagnostic cases. As in the primary analysis, the inclusion of non-diagnostic cCTA scans caused a sensible reduction of overall accuracy and PPV, while maintaining a high NPV.

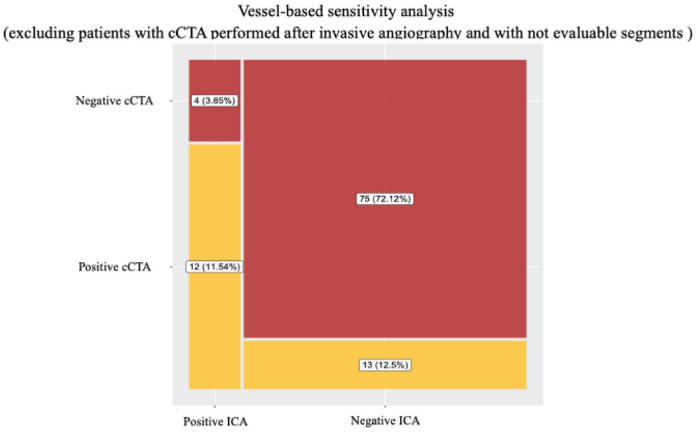

Vessel-based analysis									
No. of vessels	No of TN findings	No of TP findings	No of FN findings	No of FP findings	Sensitivity	Specificity	NPV	PPV	Accuracy
104	75	12	4	13	75 (48-93)	85 (76-92)	95 (87-99)	48 (28-69)	84 (75-90)

TN = true negatives, TP = true positives, FN = false negatives, NPV = negative predictive value, PPV = positive predictive value.

Supplementary Figure 3. Diagnostic performance of cCTA (vessel-based analysis excluding non-evaluable segments) in the sensitivity cohort (with exclusion of patients in whom cCTA was performed after ICA). Performance metrics were comparable to the primary analysis, with high NPV and acceptable accuracy after excluding non-evaluable coronary segments.

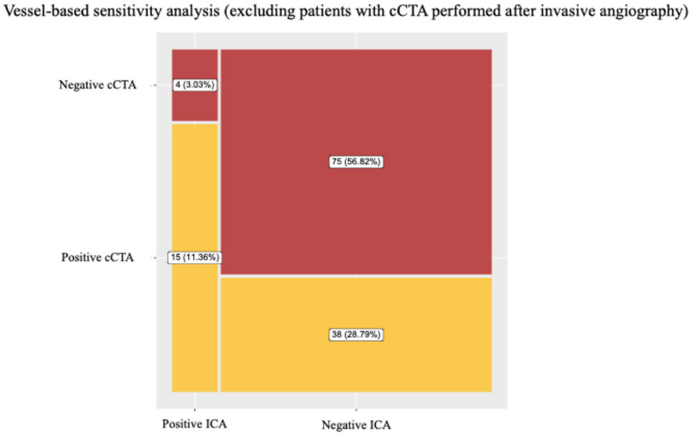

Vessel-based analysis									
No. of vessels	No of TN findings	No of TP findings	No of FN findings	No of FP findings	Sensitivity	Specificity	NPV	PPV	Accuracy
132	75	15	4	38	79 (54-94)	66 (57-75)	95 (87-99)	28 (17-42)	68 (59-76)

TN = true negatives, TP = true positives, FN = false negatives, NPV = negative predictive value, PPV = positive predictive value.

Supplementary Figure 4. Diagnostic performance of cCTA (vessel-based analysis including non-evaluable segments) in the sensitivity cohort (with exclusion of patients in whom cCTA was performed after ICA). As in the full cohort, inclusion of non-evaluable vessels diminished PPV and accuracy, reinforcing the importance of careful interpretability criteria in clinical application.
